# Serum Levels of IL-6 and TNF-α, Salivary Morning Cortisol and Intensity of Psychological Stress in Patients with Allergic Contact Hand Dermatitis and Healthy Subjects

**DOI:** 10.3390/life15030351

**Published:** 2025-02-24

**Authors:** Nives Pondeljak, Liborija Lugović-Mihić, Blaženka Ladika Davidović, Dalibor Karlović, Milena Hanžek, Marijana Neuberg

**Affiliations:** 1 Dermatovenereology Department, General Hospital “Dr. Ivo Pedišić”, Sisak, Josipa Jurja Strossmayera 59, 44000 Sisak, Croatia; 2 Dermatovenereology Department, University Hospital Center Sestre Milosrdnice, Vinogradska 29, 10000 Zagreb, Croatia; liborija@sfzg.hr; 3 School of Dental Medicine, University of Zagreb, Gundulićeva 5, 10000 Zagreb, Croatia; 4 Department of Oncology and Nuclear Medicine, University Hospital Center Sestre, Milosrdnice, Vinogradska 29, 10000 Zagreb, Croatia; blazenka.ladika.davidovic@kbcsm.hr; 5 Psychiatry Department, University Hospital Center Sestre Milosrdnice, Vinogradska 29, 10000 Zagreb, Croatia; dalibor.karlovic@gmail.com; 6 School of Medicine, Catholic University of Croatia, Ilica 242, 10000 Zagreb, Croatia; 7 Department of Clinical Chemistry, University Hospital Center Sestre Milosrdnice, Vinogradska 29, 10000 Zagreb, Croatia; milenanjegovan13@gmail.com; 8 Department of Nursing, Varaždin University Center, University of the North, Jurja Križanića 31b, 42000 Varaždin, Croatia; marijana.neuberg@unin.hr

**Keywords:** allergic contact dermatitis, psychological stress, IL-6, TNF-α, quality of life

## Abstract

Introduction: Allergic contact dermatitis (ACD) is a form of late hypersensitivity reaction of skin contact with allergens. As an inflammatory skin disease, ACD has a negative impact on the quality of life and there is a need to elucidate the etiopathogenetic factors of the disease, whereby using the psychoneuroimmunological (PNI) approach can be helpful. Psychological stress (PS), as a component of PNI, leads to aggravation of the contact hypersensitivity reaction. In response to the perception of PS, cortisol secretion is enhanced by activation of the hypothalamic–pituitary–adrenal (HPA) axis. Furthermore, the pro-inflammatory cytokines interleukin 6 (IL-6) and tumor necrosis factor alpha (TNF-α) play a role in activating the HPA axis as well as initiating and maintaining inflammatory responses. Recent studies show that IL-6, IL-1, and TNF-α values are increased in the serum of patients with contact dermatitis, as well as in keratinocyte cell culture. Methods: The study examined the association of PNI factors (serum IL-6 and TNF-α, stress intensity with a Perceived Stress Scale (PSS) questionnaire, quality of life of dermatology patients with a Dermatology Life Quality Index (DLQI)) with the disease severity evaluated using the Hand Eczema Extent Score (HEES) and the duration of disease in hand ACD patients. Results: Patients with hand ACD had higher PSS (*p* = 0.001) than healthy people, with no difference in IL-6 and TNF-α. Higher DLQI was associated with higher HEES and PSS (*p* = 0.002 and *p* < 0.001) and these were the only predictors of DLQI. The duration of the disease was not related to the investigated factors. Conclusion: This study is the first so far, to our knowledge, in which a detailed analysis of PNI factors in patients with hand ACD was conducted. The results show that patients with ACD have higher PS intensity, which can chronically indicate changes in the balance of the HPA axis and indirectly affect the quality of life and disease severity of this disease. The results of the research provide more knowledge about hand ACD and contribute to and emphasize the importance of a multidisciplinary approach to treatment, thus improving the quality of life of these patients.

## 1. Introduction

**Allergic contact dermatitis (ACD)** is a delayed hypersensitivity skin reaction caused by contact with allergens, negatively impacting the quality of life. Understanding its etiopathogenetic factors is essential, and the psychoneuroimmunological (PNI) approach may offer valuable insights. Psychological stress, as a key component of PNI, can exacerbate contact hypersensitivity reactions. When stress is perceived, the hypothalamic–pituitary–adrenal (HPA) axis is activated, leading to increased cortisol secretion. Stress, within the PNI framework, is defined as a state where an individual perceives a mismatch between situational demands and their biological, psychological, or social resources. Stress can be categorized as acute or chronic, avoidable or unavoidable. Chronic stress elevates cortisol levels, shifting the immune balance toward an intensified T helper cell (Th2) and cytotoxic lymphocyte (Tc) response [1,2,3]. Research suggests that while chronic stress increases total daily cortisol levels, the imbalance in the HPA axis disrupts the physiological circadian rhythm and cortisol concentration fluctuations observed during acute stress. Initially, chronic stress may hyperactivate the HPA axis, but prolonged exposure to stressors and exhaustion of adaptive mechanisms can eventually suppress its activity. The skin, functioning as a peripheral neuroendocrine organ, regulates its local homeostasis through hormones, neurotransmitters, neuropeptides, and cytokines secreted by various cells such as keratinocytes, dermal fibroblasts, and immune cells. These molecules mediate pro-inflammatory and anti-inflammatory effects [4].

Skin cells express numerous receptors for hormones and neurotransmitters, such as those for androgens, estrogens, cortisol, and corticotropin-releasing hormone (CRH), which modulate the skin’s response to psychological and environmental stressors [4]. Under normal conditions, keratinocytes in healthy skin do not express major histocompatibility complex type II (MHC-II) or intercellular adhesion molecule 1 (ICAM-1). However, these molecules can be expressed following pathological stimuli, stressors, or exposure to pro-inflammatory cytokines, such as tumor necrosis factor-alpha (TNF-α) [5]. This intricate network supports the concept of the skin as an autonomous immune compartment, with keratinocytes playing a crucial role. Research indicates that the skin possesses a peripheral neuroendocrine control mechanism analogous to the central HPA axis [6]. Locally, the skin features a corticotropin-releasing hormone (CRH)-proopiomelanocortin (POMC)-adrenocorticotropic hormone (ACTH)-cortisol axis, essential for stress responses. Epidermal keratinocytes and dermal fibroblasts secrete CRH, which binds to CRH receptors (CRH-R1), stimulating the production of POMC. Degradation of POMC produces hormones like ACTH, melanocyte-stimulating hormone (MSH), and β-endorphin, which bind to their respective receptors, leading to increased secretion of cortisol and corticosterone [6,7,8]. These glucocorticoids influence immune responses and inflammatory skin diseases [6,7,8]. Research using in vitro keratinocyte cultures shows that cortisol synthesis involves the enzyme 11β-hydroxysteroid dehydrogenase type 1 (11β-HSD1), which is upregulated by interleukin 1 beta (IL-1β) and TNF-α [9].

Certain allergens directly activate the innate immune system by stimulating toll-like receptors (TLRs), leading to increased IL-6 and TNF-α secretion by keratinocytes [10]. Under high stress, keratinocytes and mast cells secrete elevated levels of IL-6, which can cross the blood–brain barrier to activate the central HPA axis, creating a feedback loop of negative stress effects [11]. Additionally, IL-6 and TNF-α play a role in activating the HPA axis while initiating and sustaining inflammatory responses. Recent studies show increased serum levels of IL-6, IL-1, and TNF-α in patients with ACD, as well as in keratinocyte cultures.

Despite its importance, few studies in scientific databases (e.g., PubMed, Web of Science, Scopus) focus on this comprehensive PNI approach to inflammatory skin diseases. Notably, no studies simultaneously examine the psychological, neuroendocrine, and immune components in ACD patients [4,5,6].

## 2. Subjects

In this study, 81 respondents initially participated; however, 78 were ultimately included in the analysis, as 3 participants were excluded due to the presence of blood in their saliva samples, which made analysis impossible. Among the 78 subjects, 59 were patients with hand ACD, and 19 were healthy individuals who comprised the control group. Patients aged between 18 and 55 years with a diagnosis of chronic ACD of the hands were included in the study. The clinical presentation was limited to skin changes on the hands, with a positive and relevant epicutaneous patch test result for the standard allergen series [12]. Previous research has shown that physiological changes in HPA axis function occur in older individuals, potentially leading to falsely lower salivary cortisol levels. As a result, patients older than 55 years were excluded from the study [12]. The control group consisted of 19 randomly selected healthy individuals who met the inclusion criteria: aged 18 to 55 years, nonsmokers, non-alcohol consumers, with no history of inflammatory or autoimmune diseases, inflammatory skin conditions, or a positive response to the epicutaneous patch test for standard allergens.

Exclusion criteria for all participants were as follows: use of systemic antimicrobial drugs, antihypertensives, antineoplastic therapies, psychoactive medications, corticosteroids, cytokines, or other immunosuppressive drugs within one month prior to inclusion in the study, use of high doses of commercial probiotics (in the form of tablets or capsules), receipt of any vaccine less than 28 days before inclusion in the study, pregnancy or use of oral contraceptives, application of local antibiotics or corticosteroids within seven days before inclusion, presence of systemic inflammatory or autoimmune diseases, history of neoplasms, previously diagnosed psychiatric illnesses, diseases of the oral mucosa, smoking (due to the effects of nicotine on salivary cortisol levels) [13].

The research protocol was thoroughly explained to all participants, and they were included in the study after providing written informed consent.

## 3. Materials and Methods

After providing informed consent to participate in the research, all patients were examined by a specialist dermatovenerologist-allergologist, who collected detailed anamnestic data (with an emphasis on the duration of the skin disease) and evaluated disease severity. Objective values of PS intensity were tested by taking a morning saliva sample for analysis of cortisol levels, using the “spitting” method. All participants were asked to provide a saliva sample first, followed by the collection of serum samples. This order was chosen to avoid venipuncture-induced stress, which could influence salivary cortisol results. Taking into account the circadian rhythm of cortisol secretion, its highest concentrations are expected in the morning hours when sampling was performed (between 6 and 8 a.m., after waking up, free cortisol levels rise from 50% to 75%). In the “spitting” method, the subject collected saliva in the mouth for 5 min, and then spat it out into a graduated test tube (Salivette) (approximately 2.0 to 2.5 mL of saliva). No agents were used to stimulate saliva secretion in the subjects. The presence of blood in saliva was tested by visual reading, and saliva samples that contained blood were excluded from the study (3 samples to be exact). In the research, the ELISA method was used to analyze the value of salivary cortisol, using the Cortisol Saliva ELISA kit from EUROIMMUN^®^, Lübeck, Germany. Furthermore, patients completed the Perceived Stress Scale (PSS) and Dermatology Life Quality Index (DLQI) questionnaires. The Hand Eczema Extent Score (HEES), a validated measurement scale, was used to determine the severity of hand ACD, primarily because of its simplicity and correlation with the DLQI questionnaire, which was also used in this study [14]. The scoring system evaluates the persistence of skin changes across anatomical sites. One point is assigned to each site with skin changes, with special scoring for the dorsum and palm: 0 points for no changes, 2 points for involvement of less than two-thirds of the surface, and 4 points for involvement of more than two-thirds. The maximum possible score for each hand is 37, with a total score of up to 74 points for both hands. Based on the results, disease severity is categorized as follows: very mild (HEES 1–3), mild (HEES 4–5), moderate (HEES 6–12), severe (HEES > 13) [14].

Stress intensity was subjectively assessed using the PSS questionnaire [15,16,17]. The PSS consists of 10 Likert-scale questions (0–4). The total score reflects stress levels as follows: low stress (PSS 0–13 points), moderate stress (PSS 14–26 points), high stress (PSS 27–40 points). All participants completed the PSS questionnaire, which assesses stress experienced during the past month. The reliability of the PSS is high, with Cronbach’s alpha ranging from 0.84 to 0.86 [16].

Although many generic dermatological quality-of-life instruments exist, none are specifically designed for patients with hand ACD. For this reason, the validated DLQI questionnaire was used in this study [18,19]. The DLQI is designed for dermatological patients aged 16 and older and includes 10 questions assessing the impact of the skin condition on various aspects of daily life over the past week. Respondents choose answers that indicate the degree of impact (“very much,” “a lot,” “a little,” “not at all,” or “not relevant to me”). The total score is the sum of the responses, with higher scores indicating a greater negative impact of the skin condition on quality of life. DLQI 0–1 shows no impact, DLQI 2–5 shows small impact, DLQI 6–10 shows moderate impact, DLQI 11–20 high impact, and DLQI 21–30 extremely large impact. The reliability of the DLQI ranges from 0.70 to 0.90 across various studies [19].

The determination of immune indicators included the measurement of IL-6 and TNF-α cytokine concentrations in serum using the chemiluminescence method. Analyses were performed using an IMMULITE^®^ 1000 analyzer (Siemens Healthineers, Erlangen, Germany) and a FACSCalibur™ analyzer (Becton Dickinson, Franklin Lakes, NJ, USA). Reference values for the measured cytokines were as follows: IL-6: 0–7 pg/mL, TNF-α: 0–8.1 pg/mL (IMMULITE^®^ 1000 and FACSCalibur™).

## 4. Statistical Analysis

The Shapiro–Wilk test was used to determine whether the data followed a normal distribution. Differences in levels of salivary cortisol, serum IL-6 and TNF-α, stress intensity, and DLQI scores between patients with hand ACD and healthy controls were assessed using the Mann–Whitney test. The effect size, as a measure of the magnitude of differences between the groups, was calculated using the formula r = Z/√N for the Mann–Whitney test or r = √(t^2^/(t^2^ + df)) for *t*-tests. Spearman’s correlation was used to evaluate relationships between morning salivary cortisol levels, serum IL-6 and TNF-α, stress intensity, quality of life, disease severity, and duration, as the data did not follow a normal distribution. For positive correlations, linear regression analysis was additionally performed. The following criteria were used to interpret effect sizes: 0.14–0.36 (small effect size), 0.36–0.50 (moderate effect size), >0.50 (large effect size). The Kruskal–Wallis test was employed to compare parameters between groups formed after patient categorization, followed by the Mann–Whitney post hoc test with Bonferroni correction. The effect size for the Kruskal–Wallis test was calculated using the formula ε^2^ = χ^2^/(n − 1). Interpretation of effect size squared (r^2^) used the following thresholds: 0.14–0.36 (small effect size), 0.36–0.50 (moderate effect size), >0.50 (high effect size). All analyses were performed using IBM SPSS Statistics 22 (IBM Corp., Armonk, NY, USA).

## 5. Results

**1.**  **Basic information about the sample and parameters**

For this study, 81 subjects were initially examined (61 patients with hand ACD and 20 healthy controls). Three subjects were excluded from the study due to the presence of blood in their saliva samples, which rendered analysis impossible. The final sample consisted of 78 subjects, including 59 patients with hand ACD and 19 healthy controls. By gender, 73% of the participants were female. The median age was 38 years, with an interquartile range of 28.2–53 years, and the average age was 40.2 years. Descriptive statistics for the sample are presented in Table 1 and Table 2. There was no statistically significant difference in the gender distribution between the two groups. The median duration of chronic hand ACD among the 59 patients was 3 years, with a range of 1 to 12 years.

Based on the frequency of positive allergens identified in epicutaneous patch testing, the three most common allergens in patients with hand ACD were: nickel sulfate (40.68% of patients), cobalt chloride (30.51% of patients), thimerosal (13.56% of patients). Additionally, 35.69% of patients tested positive for more than one allergen.

**2.**  **Levels of serum IL-6 and TNF-α and intensity of psychological stress in patients with allergic contact hand dermatitis compared to healthy subjects**

In our previously published research findings, patients with hand ACD had statistically significantly lower salivary morning cortisol levels and higher PSS scores compared to healthy subjects [20]. However, the levels of cytokines IL-6 and TNF-α in the serum did not differ significantly between the groups and were not associated with HEES or DLQI scores.

**3.**  **Examining the relationship between the serum levels of IL-6 and TNF-α, and the intensity of psychological stress in relation to the severity of the clinical picture and quality of life patients with allergic contact hand dermatitis**

Statistical analysis revealed that the DLQI score of patients with chronic hand ACD was significantly associated with the HEES, reflecting disease severity, and the PSS score, which measures psychological stress intensity (r = 0.399 and 0.492; *p* = 0.002 and *p* = 0.001, respectively; Table 3). This association is moderate, linear, and positive. Therefore, as the HEES and PSS scores increase, the DLQI score also increases, indicating greater impairment of dermatological quality of life.

According to our research on the relationship between disease severity, perceived stress, and quality of life, multiple linear regression analysis showed that HEES (hand ACD disease severity) and PSS (psychological stress intensity) were the only significant predictors of quality of life when controlling for age, sex, disease duration, salivary morning cortisol levels, and IL-6 and TNF-α levels in serum. The independent contribution of psychological stress intensity to the variability in dermatological quality of life was 25%, while the contribution of hand ACD disease severity was 17% (Table 4).

In this study, an additional comparison was made between groups formed by categorizing patients with hand ACD based on the results of the measured parameters. According to the statistical analysis, specific groups were identified.

**Figure 1:**
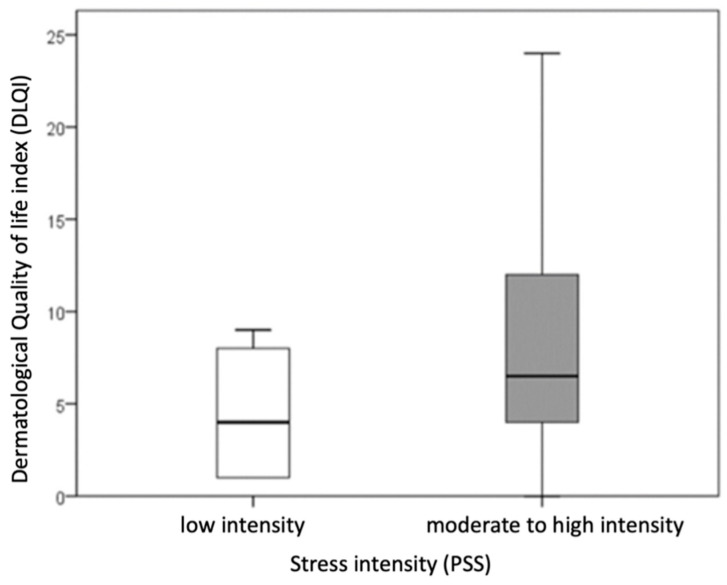
Comparison of impaired quality of life of patients with hand ACD between groups divided according to stress intensity. (rectangle plot shows median, rectangle edges interquartile range, whiskers of minimum and maximum values).

Patients with hand ACD were divided into two groups based on stress intensity: those with low stress intensity (PSS scores 0–13) and those with moderate to high stress intensity (PSS scores ≥ 14). Statistical comparison of these groups revealed a significantly higher DLQI score (indicating a more impaired dermatological quality of life) in patients with moderate to high stress intensity compared to those with low stress intensity (*p* = 0.021; r = −0.303) (Figure 1).

No significant difference was observed in the duration of the disease between patients with low stress intensity and those with moderate or high stress intensity. Due to the small number of patients (n = 4) with high stress intensity, this group was combined with the moderate stress category.

Based on disease severity, patients were categorized into groups of very mild and mild disease (HEES 1–5), moderate disease (HEES 6–12), and severe disease (HEES ≥ 13). After dividing patients with hand ACD according to disease severity (HEES), the results showed that the defined groups differed statistically significantly in salivary morning cortisol levels and the impact on dermatological quality of life (DLQI score).

**Figure 2:**
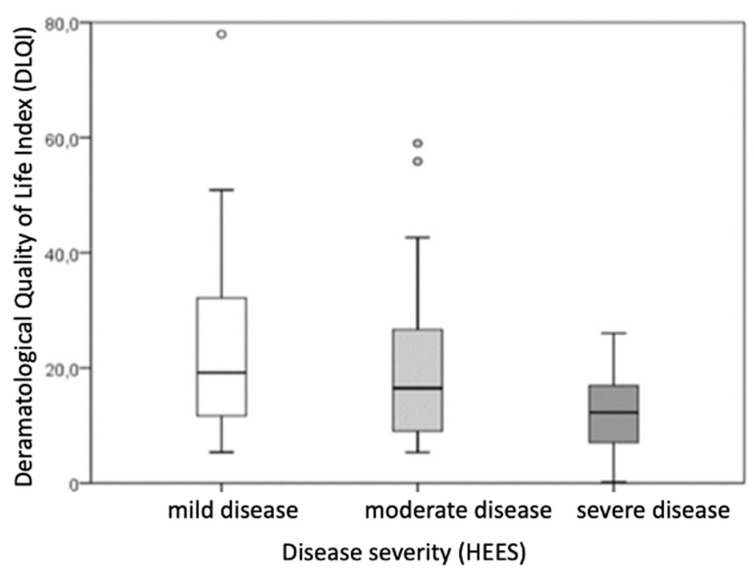
Comparison of quality of life between groups divided according to the severity of the clinical picture of patients with hand ACD. (rectangle plot shows median, rectangle edges interquartile range, whiskers of minimum and maximum values, circles of protruding values).

The DLQI score differed significantly between the groups (*p* = 0.003), with the difference being particularly significant (*p* ≤ 0.001) in comparisons between the mild group (HEES 1–5) and the severe group (HEES ≥ 13) (Table 5, Figure 2). Patients with higher HEESs had higher DLQI scores, indicating a greater impact on dermatological quality of life.

**Figure 3:**
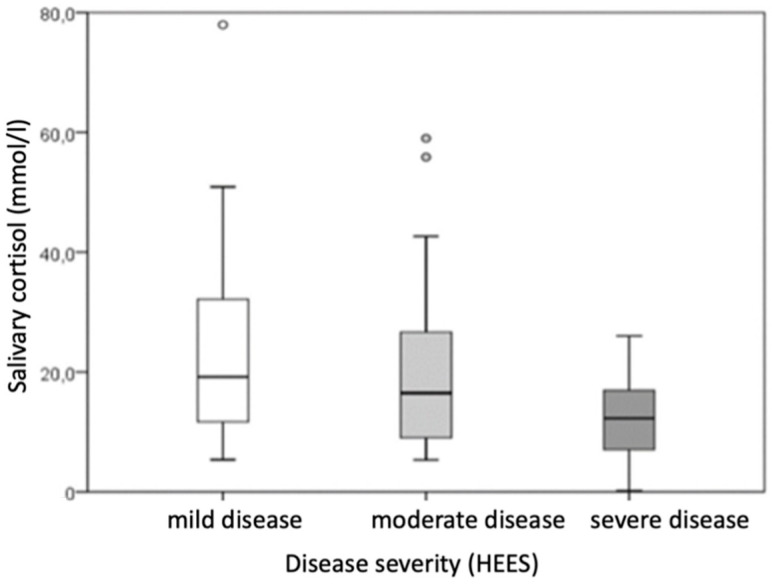
Comparison of the morning salivary cortisol level between groups divided according to the disease severity of patients with hand ACD. (rectangle plot shows median, rectangle edges interquartile range, minimum and maximum value whiskers, outlier circles).

Morning salivary cortisol levels also varied across the disease severity categories (*p* = 0.047), but pairwise comparisons between specific categories did not reach statistical significance (Table 5, Figure 3).

Based on dermatological quality of life, the sample was divided into three groups: those whose hand ACD had no impact or a very small impact on quality of life (DLQI score 0–5), those with a moderate impact (DLQI score 6–10), and those with a large or extremely large impact on dermatological quality of life (DLQI score ≥ 11).

**Figure 4:**
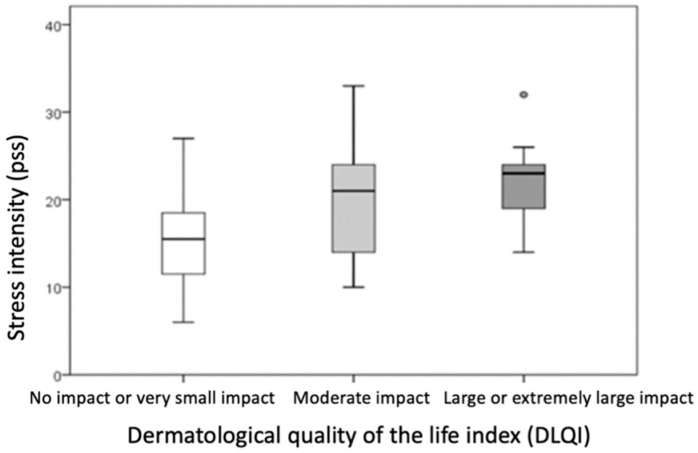
Comparison of PS intensity between groups divided according to dermatological quality of the life of patients with hand ACD. (rectangle plot shows median, rectangle edges interquartile range, whiskers of minimum and maximum values, circles of protruding values).

**Figure 5:**
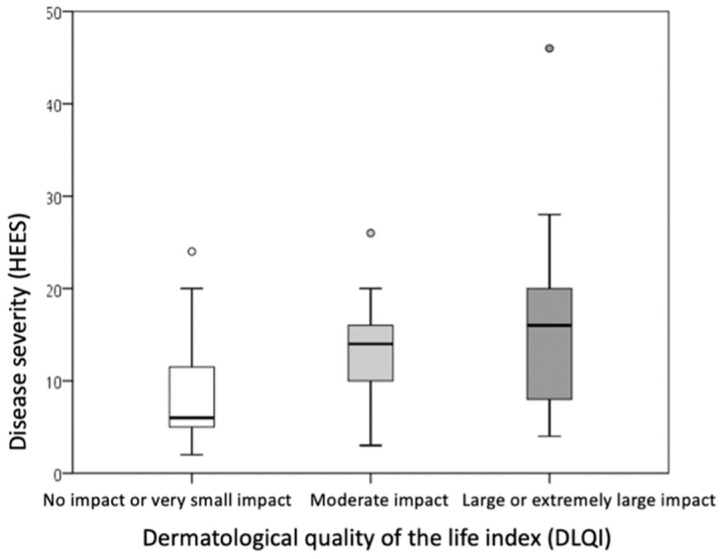
Comparison of disease severity between groups divided according to dermatological quality of the life of patients with hand ACD. (rectangle plot shows median, rectangle edges interquartile range, whiskers of minimum and maximum values, circles of protruding values).

Comparison of the groups showed that a higher DLQI score was associated with a higher PSS score (indicating higher perceived stress intensity, *p* = 0.009) and a higher HEES (indicating a more severe clinical picture, *p* = 0.015) (Table 6, Figure 4 and Figure 5). The differences were statistically significant (*p* ≤ 0.016) when comparing PSS and DLQI results between the group with no or minor impairment of dermatological quality of life (DLQI score 0–5) and the group with high or extremely high impairment of dermatological quality of life (DLQI score ≥ 11).

## 6. Discussion

To the best of our knowledge and based on data from the available literature, this is the first study to simultaneously analyze factors from all PNI domains (psychological, immunological, and endocrine) in the same patients with ACD. This is significant given the evidence that PNI factors impact many skin diseases and play a crucial role in the development, chronicity, and therapeutic response in dermatological patients [1,21].

In this study, as psychoneuroendocrine components of the PNI approach to patients with hand ACD, we examined salivary cortisol levels (considered a key objective biomarker of psychological stress and HHA axis activity) and the PSS questionnaire (as a subjective indicator of psychological stress intensity). The immunological aspects of the PNI approach included an assessment of serum IL-6 and TNF-α levels. Certain PNI factors examined in our study demonstrated a significant impact on the severity of hand ACD and the quality of life (QoL) of these patients. According to our findings, both analyzed factors of the psychoneuroendocrine component (cortisol and the PSS questionnaire) were significantly altered in hand ACD patients compared to healthy individuals. This indicates changes in HHA axis functioning, which may have important implications for the clinical approach and treatment of these patients.

It is important to note that the duration of stress may also influence pro-inflammatory biomarkers. Acute stress, through activation of the HHA axis and transiently elevated cortisol levels, can suppress IL-6 and TNF-α levels. In contrast, prolonged or chronic stress disrupts the HHA axis balance, and due to cortisol resistance, IL-6 and TNF-α levels may paradoxically increase [22]. Elevated levels of TNF-α and IL-6 have also been linked to depression, as they exacerbate and perpetuate neuroinflammatory processes, impairing brain function. For instance, studies on patients experiencing major depressive episodes have demonstrated a positive correlation between the severity of depressive symptoms, IL-6 concentrations, and cortisol levels [23,24]. In our study, despite the significantly higher stress intensity observed in patients with hand ACD compared to healthy subjects, there was no statistically significant association between elevated cytokine levels (IL-6 and TNF-α) and stress intensity, disease severity, or impaired dermatological quality of life. Furthermore, serum IL-6 and TNF-α levels did not differ significantly between patients with hand ACD and healthy controls. This suggests an absence of a systemic inflammatory response that could further influence HHA axis function.

The median PSS score in our hand ACD patients indicated moderate stress [25], but not high-intensity stress. Additionally, while these patients exhibited significantly lower morning salivary cortisol levels than healthy subjects, the median concentration of 14.94 nmol/L remained within the upper limit of the reference range for cortisol levels. Although patients with hand ACD displayed a poorer circadian cortisol response, their cortisol levels were still within physiological reference limits, indicating no complete dysfunction of the HHA axis. Therefore, it is possible that the intensity of chronic stress in our patients was insufficient to significantly impact serum IL-6 and TNF-α levels. Regarding pro-inflammatory cytokines in hand ACD, our study measured serum levels of IL-6 and TNF-α. However, the literature predominantly focuses on cytokine expression at the local level in the skin, particularly at the site of contact reactions. For example, one study analyzed the mechanisms by which specific allergens (methylchloroisothiazolinone and methylisothiazolinone) induce the production of pro-inflammatory and regulatory factors in the skin. This study observed increased expression of IL-6, FOXP3, IL-10, and TGF-β in ACD-affected skin at the contact site using qPCR and immunochemical methods [26].

Relatively few studies have examined serum cytokine levels in patients with contact hypersensitivity. For instance, Jia Q et al. compared serum IL-1β, IL-6, IL-8, and TNF-α levels in subjects exposed to trichloroethylene once, unexposed subjects, and patients with proven ACD to trichloroethylene. Their findings showed significantly higher serum levels of these cytokines in ACD patients compared to the other groups [27]. Similarly, Akhtar N et al. reported significantly elevated serum IL-6, TNF-α, IL-8, and IL-17 levels in patients with airborne ACD, alongside decreased IL-4 and IL-10 levels, compared to healthy controls [28]. Another study conducted on BALB/c mice with ACD induced by oxazolone exposure found increased serum IL-6 levels compared to healthy mice [29]. However, studies specifically investigating serum cytokine levels in hand ACD are limited, and none have reported reduced or physiological cytokine levels in ACD patients.

In our hand ACD patients, serum IL-6 and TNF-α levels were not significantly different from those of healthy controls. However, this does not rule out the possibility of elevated cytokine levels at the local level in the skin. It is known that among the immune cells involved in the etiopathogenesis of AKD, mast cells play an important role, primarily by secreting pro-inflammatory cytokines such as, for example, IL-6, TNF-α, IL-1. Considering our results, it is possible that the significant elevation of pro-inflammatory cytokines, which are crucial for contact hypersensitivity reactions, remains only at the local level at the site of skin contact with the allergen, especially in chronic disease. According to the above, the expression of an inflammatory reaction at the site of contact locally in the skin is not excluded, which was not directly examined in our research. Maybe very early in the disease process we could possibly expect a short-term increase in cytokines in the serum as well. However, according to the etiopathogenesis of ACD, in the establishment of a hypersensitivity reaction and repeated chronic exposures to the allergen (as was the case with our patients whose disease lasted for more than a year), the immune system localizes/limits the inflammation only to the places of skin contact with the allergen, and in fact it is not a chronic systemic inflammatory reaction. It is possible that the elevated level of pro-inflammatory cytokines in the serum also depends on the percentage of the skin surface that is affected by ACD lesions (extent of the clinical picture and inflammatory process), and in our patients with hand ACD, it was only a matter of localization of changes on the skin of the hands and a very small area compared to the skin of the whole body.

Patients with long-term or chronic hand ACD are potentially exposed to chronic stress due to the persistent nature of the disease and the presence of active lesions, which has been shown to affect cortisol’s physiological response and disrupt the balance of the HHA axis. The results of the PSS questionnaire in our patients confirm the existence of chronic stress, as this tool assesses the subjective experience of stress over the month preceding testing. The heightened stress intensity observed may be influenced by the chronic nature of ACD and the severity of the disease, although other life factors and individual personality traits should also be considered.

Despite lower morning cortisol levels in hand ACD patients compared to healthy subjects, these values remained within the reference range for the testing period. This suggests that, although hand ACD patients exhibited a less robust circadian cortisol response, their cortisol levels stayed within physiological limits, indicating no complete HHA axis dysfunction. Similarly to findings from Jia Q et al. [27], our results also showed no significant correlation between disease duration and the measured parameters in ACD patients. Nonetheless, the attenuated morning salivary cortisol response indirectly suggests chronically elevated total cortisol levels, which may negatively impact ACD severity (e.g., immune system dysregulation through intensified Tc responses) and impair therapeutic outcomes, potentially contributing to disease chronicity. Chronic stress disrupts HHA axis regulation, reducing cortisol production fluctuations. Chronically elevated cortisol levels shift the immune balance, intensifying Th2 and Tc responses [1,2,3]. While chronic stress increases total daily cortisol levels, the physiological acute stress response is diminished, altering the circadian rhythm. This results in higher total cortisol concentrations but an absent morning spike due to the body’s attempts to maintain balance [1]. In our study, in the already published results, hand ACD patients exhibited significantly lower morning salivary cortisol levels and significantly higher stress intensity compared to healthy controls, although no significant correlation was found between salivary cortisol levels and stress intensity [20]. Statistical analysis showed a stronger effect size for differences in stress intensity than for morning salivary cortisol levels (r = 0.425 and r = 0.392, respectively). Additionally, the median PSS score for hand ACD patients indicated moderate stress (median: 17) [20]. When examining the relationship between ACD severity and cortisol levels, more severe hand ACD was associated with lower salivary cortisol levels (*p* = 0.047), which is consistent with findings for atopic dermatitis in the literature.

Chronic hand ACD impacts the physical, material, social, and psychological aspects of life, significantly impairing health-related quality of life (QoL). The psychological burden can parallel that of other chronic conditions, such as atopic dermatitis, psoriasis, and asthma, and may lead to depression, mood disorders, low self-esteem, anxiety, and sleep disturbances [15]. Our findings demonstrated statistically significant differences in stress intensity between hand ACD patients and healthy controls, as reflected in the PSS results (*p* ≤ 0.001), aligning with prior studies. For instance, Janardhanan AK et al. examined clinical and etiological factors, including stress levels, in patients with hand eczema using the PSS. They found significantly increased stress levels in 67.7% of respondents, with high stress in 51.6% and very high stress in 16.1%. However, no differences in stress intensity were reported across subgroups of hand eczema patients, such as those with ACD versus other types [15]. Given the high stress levels in many patients with hand eczema, stress management and coping strategies should be integral to their treatment. Animal studies also highlight the significant impact of chronic ACD on stress responses. For example, research on male BALB/c mice demonstrated that chronic ACD, combined with a psychological stressor (social isolation), led to characteristic symptoms of chronic dermatitis and behavioral changes associated with fear and anxiety. These effects persisted even after stress cessation and allergen removal but improved with resocialization. Chronic stress not only intensified the contact hypersensitivity reaction but also maintained it even in the absence of the allergen, as long as the stress persisted [30].

Hand ACD patients commonly report symptoms such as itching and persistent skin changes, including rashes, which are among the most bothersome. These symptoms can disrupt sleep, cause pain and discomfort, and reduce hand functionality [31,32]. Emotional factors and chronic stress exposure often exacerbate itching and scratching through complex psychoneuroimmunological (PNI) processes and mechanisms, further impairing the patient’s quality of life (QoL) [1,31]. In one study comparing the QoL of patients with various chronic dermatoses (atopic dermatitis, contact dermatitis, occupational contact dermatitis, asteatotic dermatitis, granuloma annulare, rosacea, seborrheic dermatitis, lichen sclerosus et atrophicus, and psoriasis), lower QoL (assessed using the DLQI questionnaire) was associated with perceived serious consequences of the disease, a greater burden of symptoms, and heightened uncertainty or worry about the condition. Among the conditions studied, the most impaired dermatological QoL was observed in patients with occupational contact dermatitis [33]. Patients in this group reported low personal control over their condition and poor understanding of the disease.

In another study by Kalboussi H et al., QoL in patients with ACD was exclusively assessed using the DLQI questionnaire. The average DLQI score was 6.5, indicating a moderate impact on QoL. A more impaired QoL (i.e., a higher DLQI score) was significantly associated with accompanying atopy, total loss of work productivity, and the negative impact of the disease on daily activities. The factors most affecting QoL included itching, discomfort, and challenges in performing daily activities [34,35]. Lifestyle factors, including the living environment, also influence QoL. For instance, a comparison of patients with occupational hand ACD from large cities versus smaller communities found that lower QoL was more common in urban populations, even though they had milder clinical presentations [36]. One study assessed QoL (using the DLQI), anxiety and depression (using the Hospital Anxiety and Depression Scale, HADS), and compulsive behavior (using the Leyton Trait Scale) in patients with chronic hand ACD. The average DLQI score in this study was 11.11 ± 1.81, indicating a high impact on QoL. Patients with hand ACD scored statistically significantly higher on all questionnaires compared to healthy controls, reflecting worse QoL, more pronounced anxiety and depression, and more frequent compulsive behaviors in these patients [37].

In examining the QoL of patients with ACD, our study found that the average DLQI score indicates a moderate impact of hand ACD on QoL, aligning with the existing literature. However, an increase in the severity of the clinical presentation (HEES) and an increase in stress intensity were significantly associated with greater impairment of dermatological QoL (DLQI score). According to the results of multiple linear regression analysis, the severity of ACD and stress intensity were the only significant predictors of impaired dermatological QoL. Notably, stress intensity contributed more to the impairment (25%) than the severity of the clinical presentation (17%). These findings suggest a clear association in hand ACD patients between worse QoL, higher stress intensity (PSS score), and more severe clinical manifestations (HEESs). Patients with a greater impact on QoL exhibited both higher stress intensity and more severe ACD.

The severity of ACD is influenced primarily by the exposure/allergenicity of allergens, their concentration, and exposure conditions [38]. Skin irritation and inflammation can further exacerbate ACD and should also be considered [39,40]. Additionally, our hand ACD patients experienced a chronic disease course, including lichenification, rhagades, and fissures. It is worth noting that the HEES questionnaire used in this study exclusively assesses the quantitative presence of lesions and the percentage of skin surface involved, without accounting for the qualitative description of morphology or subjective symptoms. The localization of skin lesions on the hands contributes significantly to the deterioration of QoL in hand ACD patients. These lesions can impair daily functioning and limit the ability to perform fine motor tasks. Interestingly, in our study, the higher intensity of perceived stress had a more significant negative impact on QoL than the severity of the disease itself. Similar results were reported in a study where the stress intensity associated with living in urban environments had a greater impact on perceived dermatological QoL impairment than the actual severity of the disease [36].

Further statistical analysis of our data, after categorizing the sample by stress intensity, revealed that QoL was significantly more impaired in individuals with moderate or high stress intensity compared to those with low stress levels, as expected. While the severity of hand ACD affects stress intensity, it is important to consider other factors that influence perceived stress, such as daily life events and individual coping mechanisms. The perception of impaired QoL may also depend on additional factors, including the patient’s personality [41]. The way a person perceives their disease and copes with stress exposure is influenced by subjective characteristics and personality traits. For example, two individuals with identical disease symptoms and severity may evaluate their QoL differently due to these personal factors [42].

On the other hand, subjective but objective parameters influence dermatology patients’ compliance to treatment. Chronic stress, depressed mood, or impaired quality of life due to severity of hand ACD could make these patients less compliant with applying local treatment or accepting systemic treatment options. Also the effects of the illness on work efficiency can also make it hard for patients to apply the treatment during working hours or to comply to phototherapy, for example. Furthermore, chronic stress may elevate the cytokine IL-6 and TNF- α levels along with shifting immune system to Th2 and Tc response, as mentioned before. This could potentially lead to chronicity of the disease with a constant need for repeated dermatological therapy. In this sense, we feel that psychological intervention and therapy could be beneficial for these patients.

One of the key strengths of our research is that, to our knowledge, it is the first study to examine all components of the PNI approach in hand ACD. Furthermore, unlike the majority of studies on patients with ACD, our participants exhibited numerous positive reactions to contact allergens that were spontaneously induced without artificial exposure. Additionally, all subjects had chronic disease, making our findings more applicable to the broader population of ACD patients. Previous studies often involved subjects with reactions to only one contact allergen or artificially induced hypersensitivity reactions, such as in animal models (e.g., mice).

Another advantage of our study is the use of the HEES questionnaire, which has been proven to correlate with the DLQI questionnaire. This correlation allows for more robust results in examining these relationships, compared to studies that utilized other hand ACD severity questionnaires. The examination of multiple factors in our study supports the development of better-informed conclusions about a multidisciplinary approach to treating these patients—particularly the integration of psychiatric and psychological support—an aspect not previously explored in the ACD literature. By targeting both objective and subjective parameters, our research contributes to a broader and clearer understanding of how to approach these patients holistically. However, a limitation of our study is the lack of examination of cytokine levels (IL-6 and TNF-α) locally in the skin lesions of hand ACD patients. Investigating local cytokine levels could enhance our understanding of the etiopathogenesis of the disease and better connect PNI factors, including immune mechanisms, to the disease itself. Local cytokine analysis could also clarify the influence of HHA axis dysfunction and cortisol levels on immune responses in the skin, as well as their correlation with disease severity.

Further research could also explore the existence and extent of specific symptoms, such as itching and sleep disturbances, as well as assess patients’ personality traits, to gain deeper insights into the impact of disease severity on dermatological QoL. While our study focused exclusively on the quantitative assessment of disease severity, this approach may have limited the evaluation of the influence and correlations of qualitative factors. All patients in our study had chronic hand ACD, which inherently involved lichenification, rhagades, and fissures on the hands, but we did not investigate their extent and intensity. To further improve understanding, future studies could conduct a qualitative comparison of the QoL in hand ACD patients against healthy controls, using additional questionnaires. Such comparisons could help clarify the impact of stress and other potential contributing factors, such as personality, socioeconomic conditions, and environmental influences, on impaired QoL in this population. Although the sample size in this study was calculated prior to the beginning of the research according to sample size available in the literature, it is still a somewhat smaller sample, which may not represent the population in full. Also, further research could accentuate longitudinal follow up of these patients, even after multidisciplinary approach to treatment including psychology or psychiatric treatment. It is important to provide more extensive research in the future to define and incorporate a multidisciplinary approach to these patients.

## 7. Conclusions

Patients with hand ACD are subjectively exposed to higher levels of chronic stress and exhibit a weaker circadian (morning) response of the HHA axis compared to healthy individuals. The severity of the clinical presentation and the intensity of stress are the only statistically significant predictors of dermatological quality of life (QoL) in hand ACD patients. Greater stress intensity and more severe clinical symptoms significantly impair dermatological QoL. Among hand ACD patients, those with severe or extremely severe dermatological QoL impairment show statistically significant differences in both subjective stress intensity and disease severity. Patients with a mild clinical presentation of hand ACD have a statistically significantly lower impairment of dermatological QoL compared to those with a severe clinical presentation. Additionally, in patients experiencing moderate or high levels of stress, dermatological QoL impairment is statistically significantly worse compared to those with low subjective stress levels.

To our knowledge, this is the first study to conduct a detailed analysis of PNI factors in patients with hand ACD. For the first time, our findings provide a deeper insight into the experiences of patients with hand ACD, demonstrating significantly higher stress intensity and identifying specific parameters that contribute to dermatological QoL impairment. These findings highlight the importance of adopting a multidisciplinary approach in managing patients with hand ACD. This could include psychological support, psychiatric interventions, and therapies aimed at improving patients’ daily lives, as well as fostering collaboration between dermatologists, allergists, and other specialists (e.g., pulmonologists-allergists and otorhinolaryngologists-allergists).

A multidisciplinary approach could help hand ACD patients achieve better therapeutic outcomes and improved quality of life.

## Figures and Tables

**Table 1:** Descriptive statistics of the sample.

Variable	N	Median	Interquartile Range	Mean	SD	Min	Max
**IL-6 (pg/mL)**	78	1.40	1.40–2.00	2.08	1.71	1.4	13.4
**TNF-α (pg/mL)**	78	6.00	4.90–7.10	6.34	2.46	3.9	22.7
**Salivary cortisol (nmol/L)**	78	17.88	8.68–26.82	21.82	16.32	0.2	82.2
**PSS**	78	16.0	12.0–21.3	16.6	6.4	5	33
**DLQI**	59	6.0	3.0–10.0	6.9	4.8	0	24
**HEES**	59	10.0	5.0–16.0	11.9	7.8	2	46

N—number of subjects, SD—standard deviation, Min—minimal, Max—maximal.

**Table 2:** Gender distribution of sample.

	Group		
Gender (N)	Hand ACD	Control Group	Total Sample
**Male**	13	8	21
**%**	22.0%	42.1%	26.9%
**Female**	46	11	57
**%**	78.0%	57.9%	73.1%
**Total**	59	19	78
**%**	100%	100%	100%

**Table 3:** Correlation between parameters in serum and saliva, quality of life, weight, and disease duration in patients with hand ACD assessed by Spearman’s correlations (N = 59).

Variable		IL-6 (pg/mL)	TNF-α (pg/mL)	DQLI	HEES	PSS	Disease Duration	Salivary Cortisol
**IL-6 (pg/mL)**	r	1.000	0.180	−0.088	0.071	−0.132	0.034	0.148
	*p*	.	0.171	0.506	0.594	0.320	0.800	0.264
**TNF (pg/mL)**	r	0.180	1.000	0.066	0.062	−0.039	0.035	0.049
	*p*	0.171	.	0.620	0.639	0.770	0.793	0.711
**DQLI**	r	−0.088	0.066	1.000	0.399	0.492	−0.024	0.008
	*p*	0.506	0.620	.	0.002	<0.001	0.854	0.955
**HEES**	r	0.071	0.062	0.399	1.000	−0.048	0.020	−0.247
	*p*	0.594	0.639	0.002	.	0.716	0.881	0.060
**PSS**	r	−0.132	−0.039	0.492	−0.048	1.000	−0.204	0.053
	*p*	0.320	0.770	<0.001	0.716	.	0.121	0.692
**Disease duration**	r	0.034	0.035	−0.024	0.020	−0.204	1.000	−0.096
	*p*	0.800	0.793	0.854	0.881	0.121	.	0.469
**Salivary cortisol (nmol/L)**	r	0.148	0.049	0.008	−0.247	0.053	−0.096	1.000
	*p*	0.264	0.711	0.955	0.060	0.692	0.469	.

**Table 4:** Regression equation for predictor analysis of quality of life impairment (DLQI).

	Unstandardized Coefficient		Standardized Coefficient		Correlations		
	**B**	**SE**	**Beta**	***p***	**Zero Order**	**Partial**	**Seminpartial**
(constant)	−3.2	1.7					
PSS	0.4	0.1	0.5	<0.001	0.493	0.553	0.508
HEES	0.3	0.1	0.4	<0.001	0.396	0.476	0.414

R = 0.644, R^2^ = 0.414, corrected; R^2^ = 0.393, *p* < 0.001.

**Table 5:** Comparison of continuous parameters between groups with regard to disease severity throughout to a sample of patients with hand ACD. Values are reported as median (IQR).

Variable	Mild Disease	Moderate Disease	Severe Disease	*p*	ε^2^
IL-6 (pg/mL)	1.40 (1.40–1.70)	1.40 (1.40–2.13)	1.40 (1.40–2.00)	0.721	0.011
TNF-α (pg/mL)	5.1 (5.0–7.0)	6.9 (5.0–7.9)	6.0 (5.1–7.0)	0.508	0.023
Salivary cortisol	19.2 (8.7–37.3)	16.5 (8.8–26.8)	12.3 (6.7–17.0)	0.047	0.105
PSS	19.0 (15.0–23.0)	15.5 (12.3–22.8)	18.5 (14.0–23.8)	0.645	0.015
DLQI	4.0 (2.0–4.0)	6.0 (2.3–11.3)	9.0 (5.3–11.8)	0.003	0.203
Disease duration	2.0 (2.0–4.0)	3.0 (2.0–5.0)	3.0 (2.0–8.0)	0.322	0.006

IQR—interquartile range (25th–75th percentile), **ε^2^**—expresses measures of effect size for the difference between two means and the proportion of variance explained.

**Table 6:** Comparison of continuous variables between groups after categorization according to dermatological quality of life in patients with hand ACD.

Variable	No Impact or Very Small Impact Median (IQR)	Moderate Impact Median (IQR)	Large or Extremely Large Impact Median (IQR)	*p*	ε^2^
Disease duration (years)	3.0 (2.0–5.0)	2.0 (2.0–5.3)	4.0 (1.5–8.5)	0.812	0.007
IL-6 (pg/mL)	1.40 (1.40–2.30)	1.50 (1.40–1.93)	1.40 (1.40–1.80)	0.881	0.004
TNF-α (pg/mL)	6.3 (5.0–7.1)	6.0 (5.0–7.6)	6.0 (5.4–7.5)	0.772	0.009
Salivary cortisol (nmol/L)	15.2 (6.6–26.0)	16.5 (12.4–26.5)	12.6 (7.6–19.8)	0.124	0.072
PSS	15.5 (11.3–18.8)	21.0 (13.8–24.0)	23.0 (18.5–25.0)	0.009	0.163
HEES	6.0 (5.0–11.8)	14.0 (9.5–16.5)	16.0 (7.5–20.5)	0.015	0.146

IQR—interquartile range (25th–75th percentila), **ε^2^**—expresses measures of effect size for the difference between two means and the proportion of variance explained.

## Data Availability

The original contributions presented in this study are included in the article. Further inquiries can be directed to the corresponding author.
